# The serum follicle stimulating hormone-to-luteinizing hormone ratios can predict assisted reproductive technology outcomes in women undergoing gonadotropin releasing hormone antagonist protocol

**DOI:** 10.3389/fendo.2023.1093954

**Published:** 2023-01-30

**Authors:** Shen Zhao, Huihui Xu, Xian Wu, Lan Xia, Jian Li, Dan Zhang, Aijun Zhang, Bufang Xu

**Affiliations:** ^1^ Department of Obstetrics and Gynecology, Ruijin Hospital, Shanghai Jiao Tong University School of Medicine, Shanghai, China; ^2^ Clinical Research Center, Ruijin Hospital, Shanghai Jiao Tong University School of Medicine, Shanghai, China; ^3^ Shanghai Key Laboratory of Reproductive Medicine, Department of Histo-Embryology, Genetics and Developmental Biology, Shanghai Jiao Tong University School of Medicine, Shanghai, China

**Keywords:** embryological outcomes, GnRH antagonist (GnRH-ant) protocol, ovarian response, reproductive potential, FSH & LH

## Abstract

**Background:**

The basal follicle stimulating hormone (FSH)/luteinizing hormone (LH) ratio is a useful predictor of ovarian response. In this study, we investigated whether the FSH/LH ratios during the entire controlled ovarian stimulation (COS) can be used as effective predictors of outcomes in women undergoing *in vitro* fertilization (IVF) treatment using the gonadotropin releasing hormone antagonist (GnRH-ant) protocol.

**Methods:**

A total of 1,681 women undergoing their first GnRH-ant protocol were enrolled in this retrospective cohort study. A Poisson regression model was used to analyze the association between the FSH/LH ratios during COS and embryological outcomes. Receiver operating characteristic analysis was performed to determine the optimal cutoff values for poor responders (≤ 5 oocytes) or poor reproductive potential (≤ 3 available embryos). A nomogram model was constructed to provide a tool for predicting the cycle outcomes of individual IVF treatments.

**Results:**

The FSH/LH ratios (at the basal day, stimulation day 6 (SD6) and trigger day) were significantly correlated with the embryological outcomes. The basal FSH/LH ratio was the most reliable predictor of poor responders with a cutoff value of 1.875 (area under the curve (AUC) = 72.3%, *P* < 0.05), or of poor reproductive potential with a cutoff value of 2.515 (AUC = 66.3%, *P* < 0.05). The SD6 FSH/LH ratio predicted poor reproductive potential with a cutoff value of 4.14 (AUC = 63.8%, *P* < 0.05). The trigger day FSH/LH ratio predicted poor responders with a cutoff value of 9.665 (AUC = 63.1%, *P* < 0.05). The basal FSH/LH ratio, combined with the SD6 and trigger day FSH/LH ratios, slightly increased these AUC values and improved the prediction sensitivity. The nomogram provides a reliable model with which to assess the risk of poor response or poor reproductive potential directly based on the combined indicators.

**Conclusions:**

FSH/LH ratios are useful predictors of poor ovarian response or reproductive potential throughout the entire COS with the GnRH antagonist protocol. Our findings also provide insights into the potential for LH supplementation and regimen adjustment during COS to achieve improved outcomes.

## Introduction

The optimization and individualization of controlled ovarian stimulation (COS) is important to improve the success rate of *in vitro* fertilization-embryo transfer (IVF-ET) (1). Clinicians usually select the appropriate protocols and gonadotrophin doses or types according to the patients’ basic characteristics, such as age, anti-Müllerian hormone (AMH), antral follicles count (AFC), basal serum follicle stimulating hormone (FSH), body mass index (BMI) and the ovarian response in previous treatment cycles (2–6). Compared with the use of basal characteristics alone, integrated evaluation of multiple sensitive markers during the entire COS process will provide more accurate prediction.

Recently, concerns have been raised regarding the accuracy of serum FSH level, luteinizing hormone (LH) level and FSH/LH ratio for predicting the ovarian response and oocyte quality in IVF. FSH and LH are secreted by the pituitary gland (7). The gonadotropin FSH plays a central role in stimulating follicular growth by binding to its receptors located in the granulosa cells of the follicles (8). LH acts synergistically with FSH to stimulate follicle recruitment and promote oocyte maturation, and ovulation is triggered by an LH surge (9). Multiple studies have shown that the basal FSH and LH levels, as well as the basal FSH/LH ratio, reflect the ovarian reserve, and allow early prediction of mature oocyte yield during the GnRH agonist protocol (10–13). For instance, FSH/LH ratio inversion is a characteristic of polycystic ovary syndrome with increased LH concentrations (14), while a high basal FSH/LH ratio is predictive of higher rates of cycle cancellation, poorer ovarian response to COS or lower pregnancy rates, even with different cutoff values (2, 3, and 3.6) in different studies (10–13). During COS, the serum delta FSH levels between the starting day (basal level) and stimulation day 6 (SD6) were higher in normal responders than in hyper-responders during the GnRH-agonist protocol (15). Decreased LH concentrations during ovarian stimulation using the GnRH-agonist long protocol with rec-FSH had a negative effect on ART outcomes (16).

In recent years, the GnRH-ant protocol has become the favored choice because of its effectiveness and convenience (17). Without the prolonged suppression of pituitary FSH and LH secretion in this protocol, the potential of the FSH/LH ratio at the basal level, SD6 and trigger day to predict outcomes should be different from that for the GnRH agonist protocol. Therefore, evaluation of the ovarian response and IVF outcomes based on FSH/LH ratios during COS is important for the clinical application of the GnRH-ant protocol.

In this study, we retrospectively assessed valuable indicators to explore the potential of serum FSH/LH ratios at three representative times (basal day, SD6 and trigger day) during the COS with the GnRH-ant protocol for prediction of ovarian response and reproductive potential. This information is important to guide regimen adjustment for the GnRH-ant protocol during the entire COS to improve outcomes.

## Methods

### Patients

**Figure 1:**
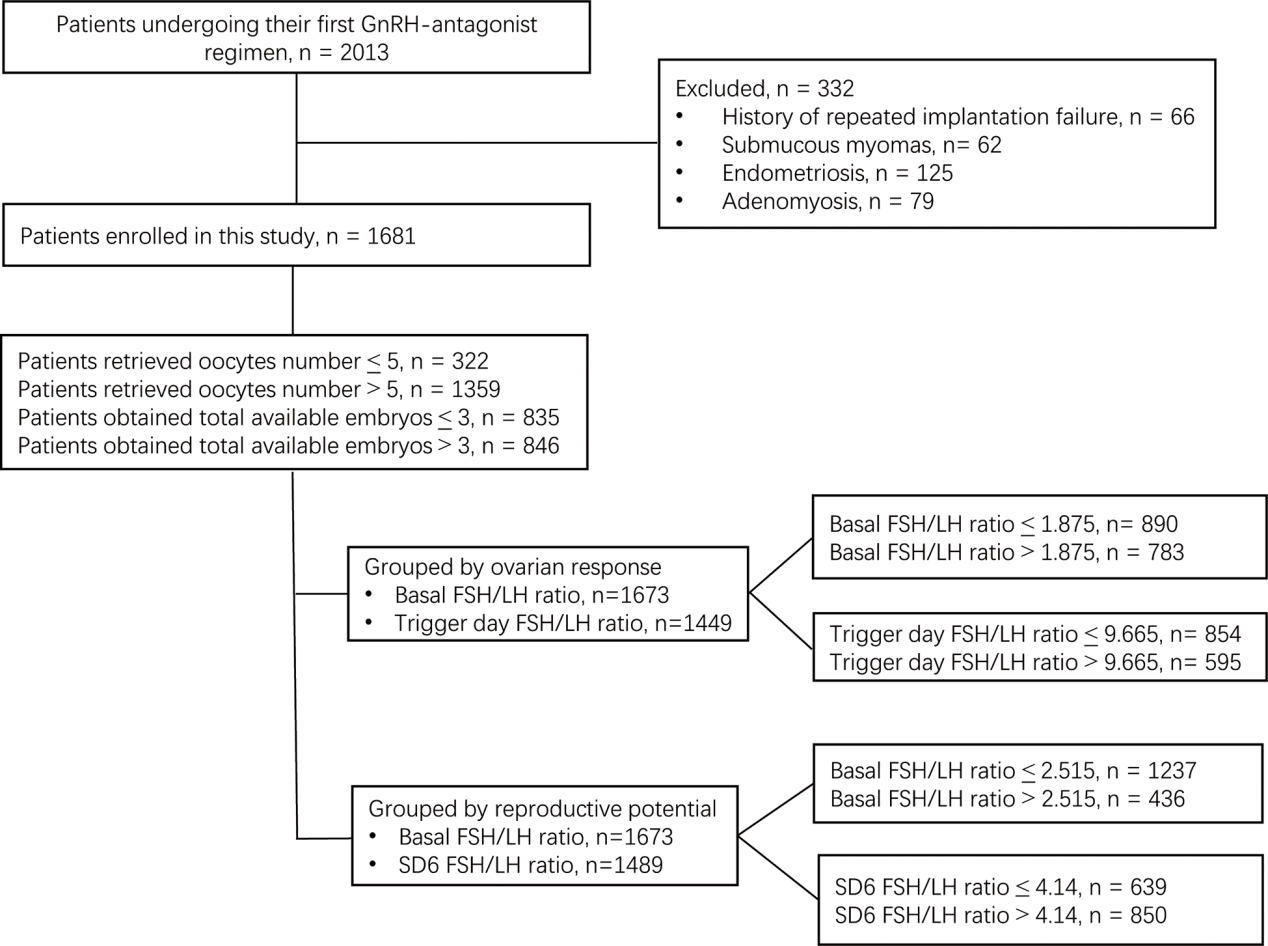
Flowchart of this study.

This retrospective cohort study was performed at the Reproductive Medical Center of Ruijin Hospital (China) from June 2017 to October 2021. A total of 2,013 patients who underwent their first ovarian stimulation following the GnRH-ant protocol were selected for eligibility according to the following inclusion criteria: (a) aged 20–40 years old with a regular menstrual cycle; (b) received fixed GnRH-ant protocol; (c) signed informed consent. The following exclusion criteria were applied: (a) known chromosomal aberration among the patients; (b) endometriosis; (c) adenomyosis; (d) submucosal myoma; (e) intramural myoma close to the endometrium or > 5 cm in size, (f) polycystic ovary syndrome. After exclusion of the patients who did not meet the inclusion criteria, a total of 1,681 patients were enrolled in this study. After exclusion due to missing basal serum FSH or LH data, 1,673 patients were included in the data analysis. After exclusion due to missing SD6 serum FSH or LH data, 1,489 patients were included in the data analysis. After exclusion due to missing trigger day FSH or LH data, 1,449 patients were included in the final data analysis (**Figure 1**).

The patients with ≤ 5 retrieved oocytes were defined as poor responders, while patients with > 5 retrieved oocytes were defined as normal responders (18). Because no consensus exists on the number of available embryos, ≤ 3 available embryos was defined as poor reproductive potential, while > 3 was defined as normal reproductive potential. The study protocol was approved by Institutional Ethics Committee of Ruijin Hospital and informed consent was obtained from all participants. The study was conducted in accordance with the Declaration of Helsinki (revised in 2013).

### Stimulation protocol

Patients’ basal FSH, basal LH, estradiol (E2), progesterone (P4) levels and AFC were measured on menstrual day 2 of the stimulation cycle. On the same day, recombinant FSH (Gonal-F, Merck-Serono SA, Switzerland) was administered, according to the baseline characteristics, with doses ranging from 112.5 to 300 IU per day. Ovarian response was monitored by routine measurement of serum FSH, serum LH, E2, P4 level and follicle scanning every 2–3 days during COS. Cetrorelix acetate (0.25 mg per day, Cetrotide, Merck-Serono, SA, Switzerland) was administered from SD6 until the day before trigger day (19). The final oocyte maturation trigger consisting of either 5,000–7,000 u hCG (Lizhu, Zhuhai, China) or 0.2 mg GnRH agonist (triptorelin acetate; France) was administered when three follicles reached a mean diameter of 17 mm. Oocyte aspiration was performed 35–36 h after the trigger day.

### IVF and embryo quality assessment

Oocytes were fertilized on the day of oocyte aspiration. Fertilization was then assessed approximately 16–18 h after insemination. Normal fertilization was confirmed by the presence of two pronuclei (PN). All fertilized oocytes were cultured in sequential media (Vitrolife, Sweden), and incubated at an atmosphere of 6% CO_2_, 5% O_2_ and 89% N_2_ at 37°C. Cleavage stage embryos at day 3 were assessed based on morphological characteristics using a standardized scoring system (19). Embryos with scores ≥ 8 were regarded as good quality embryos (20). The top two available cleavage embryos at day 3 were transferred or frozen. The surplus embryos were cultured until day 5 or 6 and the available blastocysts were transferred or frozen according to the Gardner grading system (21). The maturation rate was calculated as the percentage of metaphase II oocytes among the total number of oocytes for ICSI cycles. Fertilization rate for IVF cycle was calculated as the percentage of normal fertilized oocytes of the inseminated oocytes. The fertilization rate for ICSI cycles was calculated as the percentage of normal fertilized oocytes among the MII oocytes. Embryological outcomes were assessed based on four parameters: the number of oocytes retrieved, fertilized oocytes, good quality embryos at day 3 and total available embryos.

### Embryo transfer

One or two cleavage embryos with scores ≥ 5 on day 3, or one blastocyst with available ranking on day 5 following ovum retrieval, were transferred under transabdominal ultrasound guidance. The luteal phase was supported by 90 mg of sustained-release progesterone gel (8% Crinone; Merck-Serono, Switzerland), which was administered vaginally starting on the first day after oocyte retrieval. Biochemical pregnancy was defined as serum β-HCG > 5 mIU/ml measured 11 days after cleavage embryo transfer and 9 days after blastocyst transfer. Clinical pregnancy was defined as visualization of a gestational sac and fetal cardiac activity on transvaginal ultrasound 6 weeks after embryo transfer. All follow-up data were recorded until live birth.

### Statistical analysis

Pearson’s correlation coefficient was calculated to analyze the association of FSH/LH ratio (at basal day, SD6 and trigger day) with the embryological outcomes. A Poisson regression model was used to confirm whether the above indicators were independent determinants of the oocyte retrieval number or embryo quality. The Poisson regression model were presented as odds ratio (OR) and 95% confidence intervals (CIs). By plotting sensitivity and 1-specificity, the receiver operating characteristic (ROC) curve was constructed and the area under the ROC curve (AUC) was calculated to assess the predictive power of FSH/LH ratio for differentiating poor responders or poor reproductive potential. The cutoff value was calculated according to the maximum Youden index value, and the Youden index was calculated using the formula sensitivity + specificity -1. Analysis of variance was used to compare the differences between the subgroups according to the cutoff value. Nomogram models were constructed on the basis of determinants identified in the multivariate logistic model to predict the risk of poor ovarian responders and low ovarian potential during the cycle.

A two-sided *P*-value < 0.05 was considered to indicate statistical significance. Continuous data were reported as the mean ± standard deviation (SD) and categorical data were presented as frequencies and percentages. Mann-Whitney U-tests and Student’s *t*-tests were used to compare means for continuous data. Chi-square tests of Fisher’s exact tests were used to determine the differences between percentages for categorical data. All statistical analyses were performed using SAS software (v. 9.4) (SAS Institute Inc., USA) or R Project v.3.5.2 (The R Foundation for Statistical Computing, Vienna, Austria. http://www.r-project.org).

## Results

Among the 2,013 patients included, 1,681 patients were enrolled in this study. Of these patients, 322 had ≤ 5 retrieved oocytes, while 1,359 had > 5 retrieved oocytes. In addition, 835 patients had ≤ 3 total available embryos, while 846 patients had > 3 total available embryos (**Figure 1**).

### Relationship between FSH/LH ratios and embryological outcomes

**Table 1** shows the relationships between FSH/LH ratios during the entire COS and the embryological outcomes. After adjusting for the potential confounding factors (age, AMH, AFC, starting dose of Gn and total Gn), the basal FSH/LH ratio showed significant negative correlations with all embryological outcomes, including the number of oocytes retrieved (OR: 0.94; 95% CI: 0.93–0.96, *P* < 0.05), the number of fertilized oocytes (OR: 0.93; 95% CI: 0.91–0.95, *P* < 0.05), the number of good quality day 3 embryos (OR: 0.93; 95% CI: 0.88–0.98, *P* < 0.05) and the total number of available embryos (OR: 0.94; 95% CI: 0.91–0.96, *P* < 0.05). In addition, the SD6 FSH/LH ratio showed a significant negative association with the number of fertilized oocytes (OR:1.00; 95% CI: 0.99–1.00, *P* < 0.05) and the number of total available embryos (OR:0.99; 95% CI:0.99–1.00, *P* < 0.05). The FSH/LH ratio at trigger day showed a significant positive correlation with the number of oocytes retrieved (OR: 1.00; 95% CI:1.00–1.00, *P* < 0.05) and the number of fertilized oocytes (OR: 1.00; 95% CI:1.00–1.00, *P* < 0.05).

**Table 1:** Correlation analysis and Poisson regression analysis of factors associated with embryological outcomes.

Variable	Pearson’s correlation coefficient(γ)	Unadjusted *P*	Adjusted OR (95% CI)*	Adjusted P*
No. of oocytes retrieved				
Basal FSH/LH	-0.340	0.000	0.94 (0.93-0.96)	0.000
SD6 FSH/LH	-0.228	0.000	1.00 (1.00-1.00)	0.409
Trigger day FSH/LH	0.115	0.000	1.00 (1.00-1.00)	0.000
No. of fertilized oocytes				
Basal FSH/LH	-0.320	0.000	0.93 (0.91-0.95)	0.000
SD6 FSH/LH	-0.224	0.000	1.00 (0.99-1.00)	0.032
Trigger day FSH/LH	0.098	0.000	1.00 (1.00-1.00)	0.000
No. of day 3 good quality embryos				
Basal FSH/LH	-0.111	0.000	0.93 (0.88-0.98)	0.007
SD6 FSH/LH	-0.073	0.005	1.00 (0.99-1.01)	0.945
Trigger day FSH/LH	0.024	0.371	–	–
No. of total available embryos				
Basal FSH/LH	-0.247	0.000	0.94 (0.91-0.96)	0.000
SD6 FSH/LH	-0.209	0.000	0.99 (0.99-1.00)	0.003
Trigger day FSH/LH	0.030	0.261	–	–

OR, odds ratio; CI, confidence interval; No., Number; SD6, Stimulation Day six.

*Adjusted for confounding factors (AMH, age, AFC, starting dose of Gn and Total Gn).

### Analysis of diagnostic accuracy of serum FSH/LH ratios

**Figure 2:**
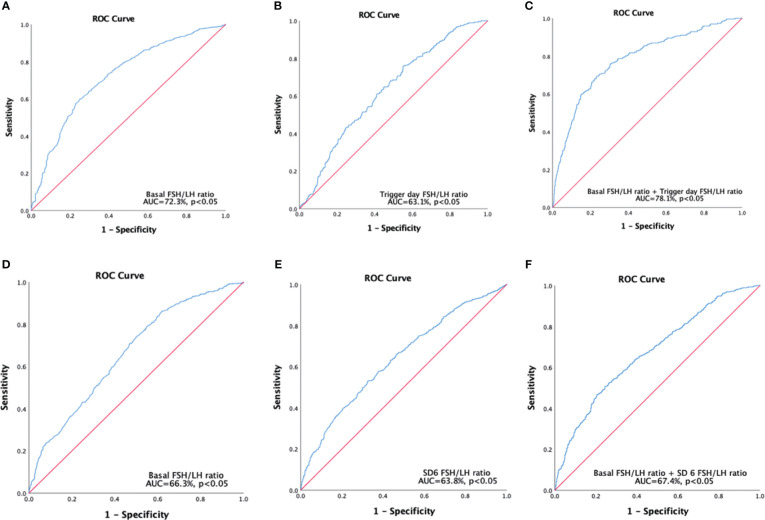
Receiver operating characteristics curves were constructed to predict poor responders **(A-C)**, and poor reproductive potential **(D-F)**. **(A)** Single variable for basal FSH/LH ratio, cutoff value=1.875. **(B)** Single variable for trigger, day FSH/LH ratio cutoff value =9.665. **(C)** Multivariable models for basal FSH/LH ratio + trigger day FSH/LH ratio; **(D)** Single variable for basal FSH/LH ratio, cutoff value=2.515. **(E)** Single variable for SD6 FSH/LH ratio, cutoff value=4.14. **(F)** Multivariable models for basal FSH/LH ratio + SD6 FSH/LH ratio. Poor responders refer to patients who retrieved oocytes ≤ 5; Poor reproductive potential refers to patients who obtained total available embryos ≤ 3.SD6: Stimulation Day six.

The basal FSH/LH ratio demonstrated significant accuracy in distinguishing poor responders from normal responders (AUC = 72.3%, *P* < 0.05) (**Figure 2A**) with a cutoff value of 1.875. The trigger day FSH/LH ratio also showed significant accuracy in distinguishing poor responders from normal responders (AUC = 63.1%, *P* < 0.05) (**Figure 2B**) with a cutoff value of 9.665. The multivariable model of the FSH/LH ratio at basal and trigger day showed a higher level of confidence in the accuracy of distinguishing poor responders from normal responders (AUC = 78.1%, *P* < 0.05) (**Figure 2C**). The AUC-ROC curve showed significant accuracy in discriminating poor reproductive potential from normal reproductive potential, with a cutoff value of 2.515 for the basal FSH/LH ratio (AUC = 66.3%, *P* < 0.05) (**Figure 2D**) and 4.14 for the SD6 FSH/LH ratio (AUC = 63.8%, *P* < 0.05) (**Figure 2E**). The multivariable model of the FSH/LH ratio at basal day and SD6 showed a higher level of confidence in the accuracy of distinguishing poor reproductive potential from normal reproductive potential (AUC = 67.4%, *P* < 0.05) (**Figure 2F**).

### Characteristics and outcomes of subgroups based on the basal FSH/LH ratio

**Table 2** shows the basic characteristics, embryological outcomes and clinical outcomes after dividing groups according to their basal FSH/LH ratio at a cutoff value of 1.875. The groups with a higher basal FSH/LH ratio (> 1.875) showed inferior basic characteristics, and the number of oocytes retrieved (8.73 ± 5.02 vs. 12.79 ± 5.58, *P* < 0.05), the number of fertilized oocytes (6.82 ± 4.22 vs. 10.07 ± 4.80, *P* < 0.05) and the number of total available embryos (3.29 ± 2.49 vs. 4.89 ± 3.32, *P* < 0.05) were significantly lower after adjusting for the confounding factors (AMH, age, AFC, starting dose of Gn and total Gn). The proportion of poor responders was also significantly higher (30.7% vs. 8.99%, *P* < 0.05) when the basal FSH/LH ratio was > 1.875. There were no significant differences in the clinical outcomes in terms of implantation rate, biochemical pregnancy rate, clinical pregnancy rate, ongoing pregnancy rate, multiple pregnancy rate, live birth rate, abortion rate and ectopic pregnancy rate between the two groups.

**Table 2:** Basic characteristic, embryological outcomes and clinical outcomes of subgroups based on the basal FSH/LH ratio cutoff value for poor responders.

	Basal FSH/LH ≤1.875	Basal FSH/LH > 1.875	*P*
N	890	783	
AMH (ng/ml)	5.32 ± 4.07	2.65 ± 2.17	0.000^a^
Age (years)	30.96 ± 3.78	32.72 ± 4.08	0.000^a^
AFC	14.46 ± 5.78	10.09 ± 4.73	0.000^a^
BMI (kg/m^2^)	22.18 ± 2.92	22.13 ± 2.88	0.583^a^
Basal FSH (IU/L)	6.98 ± 2.04	8.63 ± 3.12	0.000^a^
Basal LH (IU/L)	6.66 ± 5.57	3.18 ± 1.21	0.000^a^
Basal E2 (pg/ml)	43.92 ± 33.90	42.42 ± 37.96	0.133^a^
Basal P4 (pg/ml)	0.74 ± 1.12	0.60 ± 0.46	0.020^a^
Basal FSH/LH ratio	1.24 ± 0.41	2.95 ± 1.54	0.000^a^
Starting dose of Gn (IU)	205.65 ± 55.16	231.83 ± 56.31	0.000^a^
Stimulation duration of Gn (days)	9.76 ± 1.60	9.79 ± 1.74	0.652^a^
Total Gn (IU)	2113.91 ± 748.14	2388.91 ± 749.71	0.000^a^
EMBRYOLOGICAL OUTCOMES			
No. of oocytes retrieved	12.79 ± 5.58	8.73 ± 5.02	0.000^b^
No. of fertilized oocytes	10.07 ± 4.80	6.82 ± 4.22	0.000^b^
No. of day 3 good quality embryos	1.06 ± 1.56	0.79 ± 1.25	0.685^b^
No. of total available embryos	4.89 ± 3.32	3.29 ± 2.49	0.005^b^
Maturation rate (%)	2278/2682 (84.9%)	1711/2060 (83.1%)	0.079^c^
Fertilization rate of IVF (2PN) (%)	6531/8700 (75.1%)	3602/4772 (75.5%)	0.595^c^
Fertilization rate of ICSI (2PN) (%)	1937/2278 (85.0%)	1427/1711 (83.4%)	0.161^c^
Proportion of poor responders	80/890 (8.99%)	240/783 (30.7%)	0.000^c^
FRESH EMBRYO TRANSFER			
No. of cycles transferred	258	278	
No. of embryos transferred	410	460	
Endometrium thickness on trigger day (cm)	1.06 ± 0.16	1.04 ± 0.16	0.237^a^
P4 level on trigger day (pg/ml)	0.97 ± 0.42	0.91 ± 0.36	0.281^a^
Average number of embryos transferred	1.59 ± 0.49	1.65 ± 0.48	0.118^a^
Average score of cleavage embryos transferred	7.34 ± 1.11	7.14 ± 1.18	0.061^a^
Proportion of blastocyst embryo transfer (%)	31/258 (12.0%)	21/278 (7.60%)	0.081^c^
CLINICAL OUTCOMES			
Implantation rate (%)	141/410 (34.4%)	130/460 (28.3%)	0.051^c^
Biochemical pregnancy rate (%)	137/258 (53.1%)	131/278 (47.1%)	0.167^c^
Clinical pregnancy rate (%)	113/258 (43.8%)	110/278 (39.0%)	0.321^c^
Ongoing pregnancy rate (%)	92/258 (35.7%)	92/278 (33.1%)	0.532^c^
Multiple pregnancy rate (%)	31/258 (12.0%)	26/278 (9.40%)	0.318^c^
Live birth rate (%)	92/258 (35.7%)	92/278 (33.1%)	0.532^c^
Abortion rate (%)	16/113 (14.2%)	14/110 (12.7%)	0.754^c^
Ectopic pregnancy rate (%)	5/113 (4.40%)	4/110 (2.80%)	0.381^c^

Data is expressed as mean ± SD, or number (percentage); AMH, anti-Müllerian hormone; AFC, antral follicles count; P4, progesterone; No., Number.

a Student’s t-test or Mann-Whitney U test.

b Adjusted for confounding factors (AMH, age, AFC, starting dose of Gn and Total Gn).

c Chi-test or Fisher’s exact test.

As shown in **Table 3**, the basic characteristics of the basal FSH/LH ratio > 2.515 group were inferior compared with the group with FSH/LH ratio ≤ 2.515, and thus, the four embryological parameters were all significantly lower after adjusting for the confounding factors (*P* < 0.05), while the proportion of low reproductive potential patients was significantly higher (72.9% vs. 41.6%, *P* < 0.05). There were no significant differences in the clinical outcomes in terms of implantation rate, biochemical pregnancy rate, clinical pregnancy rate, ongoing pregnancy rate, multiple pregnancy rate, live birth rate, abortion rate and ectopic pregnancy rate between the two groups (*P* > 0.05).

**Table 3:** Basic characteristic, embryological outcomes and clinical outcomes of subgroups based on the basal FSH/LH ratio cutoff value for poor reproductive potential.

	Basal FSH/LH ≤ 2.515	Basal FSH/LH > 2.515	*P*
N	1237	436	
AMH (ng/ml)	4.73 ± 3.80	2.20 ± 1.84	0.000^a^
Age (years)	31.34 ± 3.90	33.04 ± 4.08	0.000^a^
AFC	13.57 ± 5.73	9.12 ± 4.34	0.000^a^
BMI (kg/m^2^)	22.10 ± 2.86	22.31 ± 3.01	0.327^a^
Basal FSH (IU/L)	7.25 ± 2.13	9.17 ± 3.62	0.000^a^
Basal LH (IU/L)	5.83 ± 4.94	2.76 ± 1.15	0.000^a^
Basal E2 (pg/ml)	42.90 ± 30.70	44.11 ± 47.58	0.345^a^
Basal P4 (pg/ml)	0.71 ± 0.99	0.59 ± 0.39	0.009^a^
Basal FSH/LH ratio	1.50 ± 0.55	3.58 ± 1.83	0.000^a^
Starting dose of Gn (IU)	210.02 ± 56.20	240.25 ± 54.06	0.000^a^
Stimulation duration of Gn (days)	9.74 ± 1.61	9.87 ± 1.82	0.170^a^
Total Gn (IU)	2153.90 ± 740.00	2494.42 ± 764.72	0.000^a^
EMBRYOLOGICAL OUTCOMES			
No. of oocytes retrieved	12.08 ± 5.60	7.51 ± 4.48	0.000^b^
No. of fertilized oocytes	9.51 ± 4.77	5.82 ± 3.80	0.000^b^
No. of day 3 good quality embryos	1.05 ± 1.53	0.59 ± 1.03	0.004^b^
No. of total available embryos	4.64 ± 3.17	2.71 ± 2.18	0.000^b^
Maturation rate (%)	3215/3808 (84.4%)	774/934 (82.9%)	0.243^c^
Fertilization rate of IVF (2PN) (%)	8407/11133 (75.5%)	1726/2339 (73.8%)	0.080^c^
Fertilization rate of ICSI (2PN) (%)	2724/3215 (84.7%)	640/774 (82.7%)	0.161^c^
Proportion poor reproductive potential	514/1237(41.6%)	318/436 (72.9%)	0.000^c^
FRESH EMBRYO TRANSFER			
No. of cycles transferred	390	146	
No. of embryos transferred	630	240	
Endometrium thickness on trigger day (cm)	1.06 ± 0.17	1.03 ± 0.15	0.167^a^
P4 level on trigger day (pg/ml)	0.95 ± 0.40	0.91 ± 0.37	0.569^a^
Average number of embryos transferred	1.62 ± 0.49	1.64 ± 0.48	0.545^a^
Average score of cleavage embryos transferred	7.30 ± 1.16	7.09 ± 1.13	0.063^a^
Proportion of blastocyst embryo transfer (%)	42/390 (10.8%)	10/146 (6.80%)	0.172^a^
CLINICAL OUTCOMES			
Implantation rate (%)	205/630 (32.5%)	66/240 (27.5%)	0.151^c^
Biochemical pregnancy rate (%)	200/390 (51.3%)	68/146 (46.6%)	0.332^c^
Clinical pregnancy rate (%)	165/390 (42.3%)	58/146 (39.7%)	0.589^c^
Ongoing pregnancy rate (%)	137/390 (35.1%)	47/146 (32.2%)	0.524^c^
Multiple pregnancy rate (%)	47/390 (12.1%)	10/146 (6.80%)	0.082^c^
Live birth rate (%)	137/390 (35.1%)	47/146 (32.2%)	0.534^c^
Abortion rate (%)	20/165 (12.1%)	10/58 (17.2%)	0.326^c^
Ectopic pregnancy rate (%)	8/165 (4.80%)	1/58 (1.70%)	0.452^c^

Data is expressed as mean ± SD, or number (percentage); AMH, anti-Müllerian hormone; AFC, antral follicles count; P4, progesterone; No., Number.

a Student’s t-test or Mann-Whitney U test.

b Adjusted for confounding factors (AMH, age, AFC, starting dose of Gn and Total Gn).

c Chi-test or Fisher’s exact test.

### Characteristics and outcomes of subgroups based on the SD6 FSH/LH ratio

Compared with the SD6 FSH/LH ratio ≤ 4.14 group, the SD6 FSH/LH ratio > 4.14 group had significantly poorer basic characteristics, as AMH, age, and AFC were all disadvantaged in this group, and the number of retrieved oocytes, the number of fertilized oocytes, and the total number of available embryos were all significantly lower after adjusting for the confounding factors (*P* < 0.05) (**Table 4**). The proportion of low reproductive potential patients was also significantly higher (58.8% vs. 37.9%, *P* < 0.05). There were no significant differences in the clinical outcomes in terms of implantation rate, biochemical pregnancy rate, clinical pregnancy rate, ongoing pregnancy rate, multiple pregnancy rate, live birth rate, abortion rate and ectopic pregnancy rate between the two groups (*P* > 0.05).

**Table 4:** Basic characteristic, embryological outcomes and clinical outcomes of subgroups based on SD6 FSH/LH ratio cutoff value for poor reproductive potential.

	SD6 FSH/LH ≤ 4.14	SD6 FSH/LH > 4.14	*P*
N	639	850	
AMH (ng/ml)	5.37 ± 4.24	3.08 ± 2.68	0.000^a^
Age (years)	31.27 ± 3.85	32.16 ± 4.10	0.000^a^
AFC	14.85 ± 6.20	10.90 ± 4.93	0.000^a^
BMI (kg/m^2^)	22.29 ± 3.11	22.01 ± 2.74	0.119^a^
Basal FSH (IU/L)	7.28 ± 2.31	8.11 ± 2.91	0.000^a^
Basal LH (IU/L)	6.20 ± 4.22	3.97 ± 1.99	0.000^a^
Basal E2 (pg/ml)	44.78 ± 35.09	41.41 ± 35.33	0.000^a^
Basal P4 (pg/ml)	0.67 ± 0.82	0.69 ± 0.86	0.701^a^
Basal FSH/LH ratio	1.52 ± 0.84	2.41 ± 1.51	0.000^a^
Starting dose of Gn (IU)	199.09 ± 53.53	226.35 ± 56.78	0.000^a^
Stimulation duration of Gn (days)	9.60 ± 1.76	9.86 ± 1.62	0.000^a^
Total Gn (IU)	2021.89 ± 760.11	2352.31 ± 743.66	0.000^a^
EMBRYOLOGICAL OUTCOMES			
No. of oocytes retrieved	12.82 ± 5.99	9.40 ± 5.11	0.005^b^
No. of fertilized oocytes	10.07 ± 5.03	7.35 ± 4.34	0.002^b^
No. of day 3 good quality embryos	1.08 ± 1.59	0.82 ± 1.32	0.768^b^
No. of total available embryos	5.01 ± 3.42	3.48 ± 2.58	0.001^b^
Maturation rate (%)	1642/1930 (85.1%)	1937/2327 (83.2%)	0.103^c^
Fertilization rate of IVF (2PN) (%)	4680/6261 (74.7%)	4258/5666 (75.2%)	0.613^c^
Fertilization rate of ICSI (2PN) (%)	1402/1642 (85.4%)	1616/1937 (83.4%)	0.109^c^
Proportion poor reproductive potential	242/639 (37.9%)	500/850 (58.8%)	0.000^c^
FRESH EMBRYO TRANSFER			
No. of cycles transferred	179	330	
No. of embryos transferred	287	541	
Endometrium thickness on trigger day (cm)	1.03 ± 0.15	1.06 ± 0.17	0.123^a^
P4 level on trigger day (pg/ml)	0.98 ± 0.42	0.93 ± 0.38	0.293^a^
Average number of embryos transferred	1.60 ± 0.49	1.64 ± 0.48	0.423^a^
Average score of cleavage embryos transferred	7.32 ± 1.11	7.18 ± 1.18	0.172^a^
Proportion of blastocyst embryo transfer (%)	27/179 (15.1%)	23/330 (7.00%)	0.003^c^
CLINICAL OUTCOMES			
Implantation rate (%)	87/287 (30.3%)	162/541 (29.9%)	0.912^c^
Biochemical pregnancy rate (%)	91/179 (50.8%)	161/330 (48.8%)	0.659^c^
Clinical pregnancy rate (%)	72/179 (40.2%)	136/330 (41.2%)	0.828^c^
Ongoing pregnancy rate (%)	58/179 (32.4%)	113/330 (34.1%)	0.692^c^
Multiple pregnancy rate (%)	18/179 (10.1%)	32/330 (9.70%)	0.897^c^
Live birth rate (%)	58/179 (32.4%)	113/330 (34.1%)	0.692^c^
Abortion rate (%)	10/72 (13.9%)	18/136 (13.2%)	0.895^c^
Ectopic pregnancy rate (%)	4/72 (5.60%)	5/136 (3.70%)	0.500^c^

Data is expressed as mean ± SD, or number (percentage); AMH, anti-Müllerian hormone; AFC, antral follicles count; P4, progesterone; No., Number.

a Student’s t-test or Mann-Whitney U test.

b Adjusted for confounding factors (AMH, age, AFC, starting dose of Gn and Total Gn).

c Chi-test or Fisher’s exact test.

### Characteristics and outcomes of subgroups based on the trigger day FSH/LH ratio

As shown in **Table 5**, there were no differences in the age and AFC of the trigger day FSH/LH ratio > 9.665 group compared with the group of trigger day FSH/LH ratio ≤ 9.665 (*P* > 0.05), while the AMH and BMI were slightly lower, and the starting dose of Gn and total Gn were significantly higher (*P* < 0.05). Thus, after adjusting for the basic confounding factors (AMH, BMI, starting dose of Gn and total Gn), the number of oocytes retrieved (11.76 ± 5.29 vs. 10.44 ± 5.95, *P* < 0.05), the number of fertilized oocytes (9.20 ± 4.58 vs. 8.14 ± 4.94, *P* < 0.05) and the number of total available embryos (4.21 ± 2.86 vs. 4.13 ± 3.14, *P* < 0.05) were significantly higher in the group of trigger day FSH/LH ratio > 9.665. In addition, the fertilization rate of IVF was slightly higher (75.7% vs. 74.1%) and the proportion of poor responders was lower (10.9% vs. 24.0%) in the trigger day FSH/LH ratio > 9.665 group (*P* < 0.05). For clinical outcomes, the endometrium thickness on the trigger day was slightly thinner in the trigger day FSH/LH ratio > 9.665 group (1.03 ± 0.15 cm vs. 1.07 ± 0.17 cm, *P* < 0.05), while the P4 level was also higher (1.00 ± 0.41 ng/ml vs. 0.90 ± 0.38 ng/ml, *P* < 0.05) in the group of trigger day FSH/LH ratio > 9.665. Thus, compared with the group of trigger day FSH/LH ratio ≤ 9.665, the implantation rate (25.2% vs. 33.9%), biochemical pregnancy rate (43.0% vs. 53.9%), clinical pregnancy rate (33.0% vs. 45.8%), ongoing pregnancy rate (26.0% vs. 38.6%) and live birth rate (26.0% vs. 38.5%) were all significantly lower (*P* < 0.05).

**Table 5:** Basic characteristic, embryological outcomes and clinical outcomes of subgroups based on the trigger day FSH/LH ratio cutoff value for poor responders.

	Trigger day FSH/LH≤9.665	Trigger day FSH/LH > 9.665	*P*
N	854	595	
AMH (ng/ml)	4.43 ± 4.05	3.70 ± 3.01	0.041^a^
Age (years)	31.88 ± 4.03	31.66 ± 4.06	0.264^a^
AFC	12.98 ± 6.38	12.22 ± 4.92	0.140^a^
BMI (kg/m^2^)	22.60 ± 3.00	21.52 ± 2.64	0.000^a^
Basal FSH (IU/L)	7.99 ± 2.93	7.36 ± 2.19	0.001^a^
Basal LH (IU/L)	5.51 ± 3.89	4.07 ± 2.05	0.000^a^
Basal E2 (pg/ml)	43.39 ± 38.09	41.08 ± 29.40	0.355^a^
Basal P4 (pg/ml)	0.66 ± 0.87	0.70 ± 0.81	0.001^a^
Basal FSH/LH ratio	1.94 ± 1.47	2.16 ± 1.13	0.000^a^
Trigger day FSH (IU/L)	16.46 ± 6.00	19.97 ± 6.45	0.000^a^
Trigger day LH (IU/L)	4.09 ± 3.27	1.17 ± 0.57	0.000^a^
Trigger day E2 (pg/ml)	3835.08 ± 2933.23	4209.53 ± 2590.50	0.000^a^
Trigger day P (pg/ml)	1.04 ± 0.70	1.32 ± 1.02	0.000^a^
Starting dose of Gn (IU)	208.41 ± 57.28	223.03 ± 55.66	0.000^a^
Stimulation duration of Gn (days)	9.63 ± 1.79	9.98 ± 1.43	0.000^a^
Total Gn (IU)	2123.07 ± 786.24	2357.98 ± 713.68	0.000^a^
EMBRYOLOGICAL OUTCOMES			
No. of oocytes retrieved	10.44 ± 5.95	11.76 ± 5.29	0.000^b^
No. of fertilized oocytes	8.14 ± 4.94	9.20 ± 4.58	0.000^b^
No. of day 3 good quality embryos	0.95 ± 1.49	0.90 ± 1.37	0.852^b^
No. of total available embryos	4.13 ± 3.14	4.21 ± 2.86	0.000^b^
Maturation rate (%)	1963/2341 (83.9%)	1467/1759 (83.4%)	0.698^c^
Fertilization rate of IVF (2PN) (%)	4873/6573(74.1%)	3968/5240 (75.7%)	0.048^c^
Fertilization rate of ICSI (2PN) (%)	1654/1963 (84.3%)	1226/1467 (83.6%)	0.588^c^
Proportion of poor responders	205/854 (24.0%)	65/595(10.9%)	0.000^c^
FRESH EMBRYO TRANSFER			
Number of cycles transferred	306	200	
Number of embryos transferred	495	329	
Endometrium thickness on trigger day (cm)	1.07 ± 0.17	1.03 ± 0.15	0.007^a^
P4 level on trigger day (pg/ml)	0.90 ± 0.38	1.00 ± 0.41	0.003^a^
Average number of embryos transferred	1.62 ± 0.49	1.65 ± 0.48	0.534^a^
Average score of cleavage embryos transferred	7.23 ± 1.16	7.24 ± 1.18	0.877^a^
Proportion of blastocyst embryo transfer (%)	29/306 (9.50%)	22/200 (11.0%)	0.578^c^
CLINICAL OUTCOMES			
Implantation rate (%)	168/495 (33.9%)	83/329 (25.2%)	0.008^c^
Biochemical pregnancy rate (%)	165/306 (53.9%)	86/200 (43.0%)	0.016^c^
Clinical pregnancy rate (%)	140/306 (45.8%)	66/200 (33.0%)	0.004^c^
Ongoing pregnancy rate (%)	118/306 (38.6%)	52/200 (26.0%)	0.003^c^
Multiple pregnancy rate (%)	35/306 (11.4%)	17/200 (8.50%)	0.287^c^
Live birth rate (%)	118/306 (38.6%)	52/200 (26.0%)	0.004^c^
Abortion rate (%)	17/140 (12.1%)	12/66 (18.2%)	0.245^c^
Ectopic pregnancy rate (%)	5/140 (3.60%)	2/66 (3.00%)	0.841^c^

Data is expressed as mean ± SD, or number (percentage); AMH, anti-Müllerian hormone; AFC, antral follicles count; P4, progesterone; No., Number.

a Student’s t-test or Mann-Whitney U test.

b Adjusted for confounding factors (AMH, BMI, starting dose of Gn and Total Gn).

c Chi-test or Fisher’s exact test.

### Predictive nomogram for poor ovarian response or poor reproductive potential

**Figure 3:**
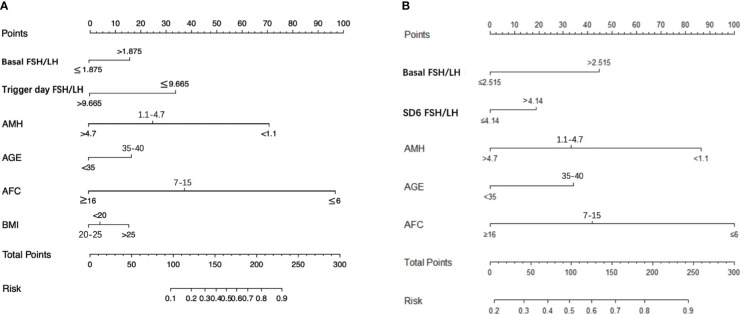
Nomogram graph to predict poor responder or poor reproductive potential for patients underwent IVF-ET. **(A)** obtained ≤ 5 oocytes, **(B)** obtained ≤ 3 available embryos during the cycle. AMH, anti-Mullerian hormone; BMI, body mass index; AFC, antral follicles count; SD6, Stimulation Day six.

As shown in **Figure 3**, nomogram models were constructed to predict the risk of poor response or poor reproductive potential, with the following variables entered into the model: age (< 35 y, 35–40 y), AMH (< 1.1 ng/ml, 1.1–4.7 ng/ml, > 4.7 ng/ml), BMI (< 20 kg/m^2^, 20–25 kg/m^2^, > 25 kg/m^2^), AFC (< 6, 7-15, >16) and FSH/LH ratio at basal, SD6 and the trigger day of COS. Using this model, the risk is calculated as the sum of the individual points identified on the point scale for each variable. The total points for each variable projected onto the lower scale indicate the risk of poor response (**Figure 3A**), and the risk for poor reproductive potential (**Figure 3B**) during this cycle.

## Discussion

In this study, we investigated the association of the FSH/LH ratios during the entire COS with ovarian response and reproductive potential at three key stages (the starting day, the sixth day and the trigger day) during treatment with the GnRH-ant protocol. For the fixed GnRH-ant protocol, patients’ hormone levels at the start of the stimulation reflect the real levels without prolonged suppression of pituitary FSH and LH secretion, while the hormone levels at SD6 reflect the levels after ovarian stimulation without downregulation. In addition, the hormone levels on the trigger day reflect the levels after short-term suppression by GnRH-ant. These three stages, which correspond to the early, middle and late stages of follicular development, represent important time-points for evaluating ovarian response during GnRH-ant protocol.

As shown in **Table 1**, the basal FSH/LH ratio showed a significant negative correlation with all embryological outcomes. In contrast, the trigger day FSH/LH ratio was positively correlated only with the number of oocytes retrieved and fertilized oocytes, and showed no correlation with the embryo quality. The SD6 FSH/LH ratio was related only to the number of fertilized oocytes and total available embryos, and showed no correlation with the number of oocytes retrieved. These findings indicated that the basal FSH/LH ratio is the most important predictor of ovarian reserve, while the FSH/LH ratios at the other two time-points (SD6 and trigger day) can be used as secondary indicators for guiding the regimen. Previous studies showed that elevated day 3 FSH/LH ratio was associated with reduced ovarian response and pregnancy rates in patients undergoing IVF with the GnRH agonist protocol (10, 11, 22, 23). Therefore, we first evaluated the potential of basal FSH/LH for prediction of ovarian response and embryo quality using the GnRH-ant protocol. Furthermore, our results suggested that the basal FSH/LH ratio is a potential indicator to predict poor responders with a cutoff value of 1.875 (AUC = 72.3%, *P* < 0.05). The basal FSH/LH ratio also showed significant ability to discriminate poor reproductive potential from normal groups with a cutoff value of 2.515 (AUC = 66.3%, *P* < 0.05).

The patients were then further divided according to the basal FSH/LH ratio cutoff value (1.875) for poor responders. The ovarian reserve parameters (age, AMH, AFC, basal FSH and basal LH) were apparently inferior in the basal FSH/LH > 1.875 subgroup, with a relatively higher starting and total dose of Gn. After correction for these confounding factors, the number of oocytes retrieved, fertilized oocytes and the number of total available embryos showed significant disadvantages in this subgroup and the incidence of poor responders remained higher. The differences in the ovarian reserve and embryo quality were more apparent between subgroups divided by the basal FSH/LH ratio (2.515). All of the results showed that the ovarian response or reproductive potential was inferior in the subgroups with higher basal FSH/LH ratio (> 1.875, or > 2.515) and that the higher basal FSH level and lower basal LH level lead to this change.

Furthermore, the SD6 FSH/LH ratio is a potential indicator to predict poor reproductive potential with a cutoff value of 4.14 (AUC = 63.8%, *P* < 0.05). The combination of the basal FSH/LH ratio and the SD6 FSH/LH ratio provided higher confidence in the prediction of poor reproductive potential with a higher AUC at 67.4%, and showing a moderate complementary value. The subgroup with a higher SD6 FSH/LH ratio (> 4.14) showed poor ovarian reserve, with a lower AMH, advanced age, higher FSH level, lower LH level and higher basal FSH/LH ratio. Compared with the starting day of the Gn stimulation, the FSH/LH ratio was higher at SD6 after 5 days of Gn treatment. This could be accounted for in several ways. First, the high FSH level at SD6 might benefit from the high starting and total dose of Gn. Second, population differences in pharmacokinetics might lead to higher FSH levels (24). Third, polymorphisms of the FSH receptor (FSHR), which is located in ovarian granulosa cells and is sensitive to exogenous rFSH (25), might play a role since such genetic variations may reduce the affinity of the receptor for serum FSH (26, 27). Therefore, patients with such polymorphisms have higher serum FSH levels due to the lower affinity of the FSH receptor. The proportion of patients with poor reproductive potential was also higher in the group of SD6 FSH/LH ratio > 4.14. Despite the higher starting and total doses of Gn, embryological outcomes were still disadvantaged after adjusting for confounding factors. Our results were consistent with a previous report that a high FSH/LH ratio in the early phase of the COS had a negative effect on oocyte quality (10).

These results provide evidence that the FSH/LH ratio at the starting day or SD6 are significant predictors of ovarian response and reproductive potential. It can be speculated that recombinant human follicle stimulating hormone (rFSH) adjustment and effective LH supplementation at the two important time-point might improve the embryological outcomes for poor ovarian responders. LH plays an important role during folliculogenesis by regulating both granulosa and theca cells. It has been hypothesized that carriers of a less bio-active LH may require higher Gn doses and/or benefit from LH activity supplementation during ovarian stimulation (28, 29). A recent study showed a higher incidence of top-quality pre-implantation embryos when LH activity was supplemented in women undergoing IVF (30). It has also been demonstrated that LH supplementation rescues the ovarian response in patients with an initial poor response to rFSH and increases the number of oocytes retrieved or the rate of clinical pregnancy (29, 31). However, the optimal LH supplementation dosage and treatment regimen remains to be established. Our study suggested that embryological outcomes using the GnRH-ant protocol can be improved by LH supplementation based on the cutoff value.

The trigger day FSH/LH ratio is also a potential indicator to predict poor responders with a cutoff value of 9.665 (AUC = 63.1%, *P* < 0.05). The combination of the trigger day FSH/LH ratio with the basal FSH/LH ratio provided higher confidence in the prediction of poor responders, with a higher AUC (AUC = 78.1%, *P* < 0.05). The subgroup with a higher FSH/LH ratio (> 9.665) showed lower ovarian reserve, with a significantly lower AMH. However, the FSH level on the trigger day was significantly higher in this subgroup, which might result from the higher starting and total doses of Gn. This could be accounted for by a higher dose of rFSH leading to higher serum FSH levels and a slightly greater oocyte yield (32, 33). Therefore, the number of oocytes retrieved, fertilized oocytes and the number of total available embryos were higher in this subgroup. The proportion of poor responders was also smaller. These results indicated that appropriately increased amount of Gn for poor responders can improve the number of oocytes retrieved and total number of available embryos. Thus, our findings indicate that the trigger day FSH/LH ratio shows complementary value for predicting the poor responders.

Based on the changes in the FSH/LH ratio during COS, we then constructed a nomogram model to predict the risk of a poor response or poor reproductive potential in women undergoing GnRH-ant protocol (**Figure 3**). For instance, for a 30-year-old woman with AMH 1.5 ng/ml, BMI 22.5, total AFC 5, basal FSH/LH ratio > 1.875, SD6 FSH/LH ratio > 4.14; trigger day FSH/LH ratio < 9.665, the risk of poor ovarian response was approximately 50%, and the risk of poor reproductive potential was approximately 75%. Thus, further studies are warranted to verify and optimize this model for accurate prediction of outcomes in women receiving the GnRH-ant regimen.

In terms of clinical outcomes, there were no significant differences in the basal and SD6 FSH/LH ratio cutoff values among the subgroups, suggesting that a higher FSH/LH ratio in the early follicle phase does not affect endometrial receptivity. However, all the clinical outcomes in the subgroup with a higher FSH/LH ratio on the trigger day were significantly inferior compared with those of the subgroup with a lower FSH/LH ratio at the same time-point, most likely due to the significantly higher P4 level and lower endometrium thickness on the trigger day. Thus, for those patients, we do not recommend fresh embryo transfer in that cycle.

The limitations of this study should be noted. First, the potential bias of the study design cannot be excluded. Second, only a small number of patients underwent fresh embryo transfer, which might affect the clinical outcomes. Therefore, a prospective study of a larger number of patients is required to confirm our findings.

In conclusion, our findings indicate that the basal FSH/LH ratio provides the most prediction of the ovarian response and embryological outcomes in women undergoing the GnRH-ant regimen. The FSH/LH ratio at SD6 and the trigger day of COS can be used as secondary indicators for guiding the regimen. The cutoff value of the FSH/LH ratio at the starting day and SD6 can be used as guide to adjust the regimen and improve clinical outcomes.

## Data availability statement

The original contributions presented in the study are included in the article/**Supplementary Material**. Further inquiries can be directed to the corresponding authors.

## Ethics statement

The studies involving human participants were reviewed and approved by Institutional Ethics Committee of Ruijin Hospital. The patients/participants provided their written informed consent to participate in this study.

## Author contributions

BX, AZ and DZ conception and design, review and final approval of the version to be published. JL analyses the data. SZ and HX draft and revise the article. XW and LX collect and analyze the data. All authors contributed to the article and approved the submitted version.

## Supplementary material

The The Supplementary Material for this article can be found online at: https://www.frontiersin.org/articles/10.3389/fendo.2023.1093954/full#supplementary-material
